# The promising role of engineered T lymphocytes in immunotherapy for high-grade serous ovarian carcinoma: a review of mechanisms, clinical landscape, and future strategies

**DOI:** 10.1186/s43046-026-00389-0

**Published:** 2026-07-27

**Authors:** Dwi Faradina, Muhammad Rusda, Muhammad Fidel Ganis Siregar, Cut Adeya Adella, Agung Putra, Arya Tjipta Prananda, Dodi Suardi, Johny Marpaung, Felix Khosasi

**Affiliations:** 1 Department of Obstetrics and Gynecology, Faculty of Medicine, Universitas Sumatera Utara, Medan, Indonesia; 2 Stem Cell and Cancer Research (SCCR) Laboratory, Stem Cell and Cancer Research, Semarang, Indonesia; 3 Lecturer, Biomedical Sciences Doctoral Program, Faculty of Medicine, Sultan Agung Islamic University, Semarang, Indonesia; 4 Department of Surgery, Faculty of Medicine, Universitas Sumatera Utara, Medan, Indonesia; 5 Department of Obstetrics and Gynaecology, Faculty of Medicine, Universitas Padjadjaran, Bandung, Indonesia; 6 Faculty of Medicine, Universitas Sumatera Utara, Medan, Indonesia

**Keywords:** CAR-T cells, TCR-T cells, Ovarian carcinoma, Immunotherapy and cancer

## Abstract

High-grade serous ovarian carcinoma (HGSC) demonstrates poor prognosis with approximately 80% recurrence rates and significant chemotherapy resistance. Conventional checkpoint inhibitors show limited efficacy (10–15%), necessitating alternative immunotherapeutic approaches. Engineered T lymphocytes, particularly CAR-T and TCR-T cells, have emerged as promising strategies for HGSC. This literature review examines the promising role of engineered T lymphocytes in immunotherapy for HGSC. A comprehensive literature search was conducted across PubMed, Scopus, and Cochrane Library databases for peer-reviewed studies published between 2015 and 2025. Search terms included “engineered T lymphocytes,” “CAR-T cells,” “TCR-T cells,” “ovarian cancer immunotherapy”. CAR-T cells demonstrate promising preclinical antitumor activity in HGSC, with high-avidity T-cell clones showing robust efficacy. Engineered T lymphocytes demonstrate promising approaches across disease contexts. In high-grade serous ovarian carcinoma, Engineered T lymphocytes orchestrate tumor cell apoptosis through coordinated granzyme/perforin and Fas/FasL signaling, enabling rapid cytotoxic elimination of multiple tumor targets.

## Introduction

Ovarian cancer is a leading gynecological malignancy with high mortality globally. According to GLOBOCAN 2022, over 324,000 new cases and 206,000 deaths occur annually worldwide. In Indonesia, it ranks second after cervical cancer with approximately 15,130 new cases and 9,673 deaths per year [1]. High Grade Serous Carcinoma (HGSC) represents 70–80% of epithelial ovarian cancers. Standard treatment entails primary debulking surgery followed by platinum-based adjuvant chemotherapy or neoadjuvant chemotherapy combined with interval debulking. While initial clinical remission is high (~ 80%), recurrence rates remain approximately 80%, particularly in advanced stages, leading to poor prognosis despite early detection [2, 3].

Immunotherapy has emerged as a fourth pillar in cancer treatment alongside surgery, chemotherapy, and radiotherapy. However, immune checkpoint inhibitors (e.g., PD-1/PD-L1 inhibitors) show limited efficacy in ovarian cancer with objective response rates around 10–15%. This has driven exploration of more precise strategies such as genetically engineered T lymphocytes expressing tumor-specific receptors. T-cell receptor (TCR)-engineered T cells and chimeric antigen receptor T cells (CAR-T) represent innovative immunotherapies. CAR-T therapy has achieved remarkable success in hematologic malignancies but faces challenges in solid tumors like ovarian cancer, including antigen heterogeneity, tumor infiltration, and immunosuppressive microenvironment [4–6]. Preclinical studies demonstrate promising antitumor activity of CAR-T targeting antigens such as MUC16 (CA-125), while TCR-T therapy targeting NY-ESO-1 has shown efficacy in murine models [7, 8]. This literature review aims to critically synthesize current knowledge on CAR-T and TCR-T cell immunotherapies in ovarian cancer, with emphasis on their mechanisms of action, clinical challenges, and future directions. This review highlights the translational potential of engineered T lymphocytes and identifies research gaps that must be addressed to advance clinical implementation.

### Search strategy and data sources

A comprehensive literature search was conducted to identify relevant studies published between January 2015 and October 2025 focusing on the mechanistic role of engineered T lymphocytes in immunotherapy for high-grade serous ovarian carcinoma (HGSC). Electronic databases searched included PubMed, Scopus, Cochrane Library, and Google Scholar. Search terms comprised MeSH headings and free-text terms: “engineered T lymphocytes”, “CAR-T cells”, “TCR-T cells”, “tumor-infiltrating lymphocytes”, “immunotherapy”, “ovarian cancer”, “high-grade serous carcinoma”. Boolean operators (AND, OR) refined the search strategy to ensure comprehensive coverage. Reference lists of key articles were screened to identify additional relevant studies.

### Inclusion and exclusion criteria

Inclusion Criteria: Studies published in peer-reviewed journals (2015–2025) examining molecular mechanisms, signaling pathways, and preclinical or clinical efficacy of engineered T lymphocytes in HGSC, written in English. Eligible study types included original research articles, mechanistic reviews, clinical trials, case reports, and preclinical studies. Exclusion Criteria: Studies were excluded if they (1) lacked mechanistic characterization or methodological detail; (2) addressed non-serous ovarian carcinomas exclusively; (3) focused on unmodified T lymphocytes; (4) did not involve engineered T lymphocyte therapy; or (5) lacked full-text availability.

### Ovarian cancer and resistance mechanisms

Ovarian cancer is a malignant disease characterized by abnormal cell proliferation within the ovarian tissue [9]. As the leading cause of mortality among female genital malignancies, ovarian cancer presents a particularly poor prognosis due to late clinical presentation, high chemotherapy resistance rates, and significant tumor recurrence incidence [10]. Approximately 80% of advanced-stage ovarian cancer patients and 20% of early-stage patients experience recurrence. Median progression-free survival (PFS) ranges from 12 to 18 months, with five-year overall survival rates of approximately 45–50%. Despite standard surgical and chemotherapeutic interventions, many patients develop recurrent disease and chemotherapy resistance, resulting in five-year survival rates of only 30–40%. Poly (ADP-ribose) polymerase inhibitors (PARPis) improve survival outcomes; however, therapeutic resistance remains a critical challenge. Chemotherapy drug resistance operates through four primary mechanisms: (1) impaired drug transport, (2) altered DNA damage repair (DDR), (3) dysregulation of cancer signaling pathways, and (4) epigenetic modifications [11]. 

### Tumor microenvironment in ovarian cancer: cellular composition and functions

The tumor microenvironment (TME) in ovarian cancer comprises a dynamic and heterogeneous network of cancer stem cells (CSCs), immune cells, stromal components, extracellular matrix (ECM), vasculature, and soluble signaling factors that collectively regulate tumor progression, metastasis, and therapeutic response. Ovarian cancer stem cells, characterized by markers such as CD44 and aldehyde dehydrogenase (ALDH), contribute to tumor initiation, self-renewal, and resistance to conventional therapies. The immune compartment includes cytotoxic CD8⁺ T cells, regulatory T cells (Tregs), and myeloid-derived suppressor cells (MDSCs), whose interactions shape antitumor immunity and immune evasion. Stromal cells, particularly tumor-associated fibroblasts (TAFs), secrete cytokines and growth factors that promote CSC maintenance and tumor progression, while the ECM provides structural support and regulates cellular adhesion, migration, and signaling. Collectively, these components establish an immunosuppressive microenvironment that facilitates CSC persistence, promotes therapeutic resistance, and impairs effective antitumor immune responses, thereby providing a strong rationale for engineered T-cell therapies targeting both CSCs and TME-mediated immunosuppressive pathways [12]. 

### T lymphocytes: development, differentiation and anti tumor immune responses

T lymphocytes are diverse cell populations maturing through positive and negative selection in the thymus, playing critical roles in cellular and humoral immunity. Two major subsets are CD4 + T helper cells and CD8 + cytotoxic T lymphocytes (CTLs). Unlike B cells, T lymphocytes recognize only protein-based antigens presented on major histocompatibility complex (MHC) molecules via T-cell receptors (TCRs). CD4 + cells recognize MHC class II complexes while CD8 + cells recognize MHC class I complexes. All T lymphocytes express TCR and the pan-T-cell co-receptor CD3, with additional co-receptors (CD4 or CD8) enabling MHC recognition. T lymphocyte identification relies on flow cytometry using CD markers [13]. TCR comprises alpha and beta chains with variable regions evolved to recognize diverse antigens through positive and negative selection during thymic maturation. A minor population, gamma-delta T cells, express delta and gamma chains and predominantly reside in mucosal epithelium, recognizing alternative antigen types. T lymphocytes express chemokine receptors (CCR5, CXCR4) on their surface, notably on CD4 + cells, serving as HIV co-receptors [13]. 

CD8 + Cytotoxic T Lymphocytes initially exist in a naïve state and require activation through interaction with professional antigen-presenting cells (APCs), particularly dendritic cells in lymphoid tissues. Activation upregulates antigen-specific TCRs and initiates effector functions. Activated CD8 + CTLs migrate to target sites and eliminate target cells through two mechanisms: (1) Fas/Fas ligand-mediated apoptosis via caspase activation, or (2) release of granzymes and perforin, which penetrate target cell membranes and activate caspases [14]. CD4 + T Helper Cells similarly require APC-mediated activation through MHC class II recognition. Activated CD4 + cells secrete cytokines initiating cellular immune responses and amplifying humoral immunity by activating B lymphocytes to produce immunoglobulins. Distinct CD4 + subsets include: (1) Th1 cells, critical for macrophage activation against intracellular pathogens via IFN-γ and TNF-α secretion, also involved in delayed-type hypersensitivity; (2) Th2 cells, essential for helminthic immunity through IL-4, IL-5, IL-13 production activating eosinophils and mast cells, also implicated in allergic diseases; and (3) Th17 cells, vital for mucosal immunity against extracellular bacteria and fungi via IL-17 A, IL-17 F, IL-22 secretion, promoting inflammation and potentially contributing to autoimmune disorders when pathologically dysregulated [14]. 

#### T lymphocyte activation mechanisms

T lymphocyte activation requires three sequential signals. Signal 1 initiates with TCR recognition of antigen-MHC complexes presented by antigen-presenting cells (APCs), triggering intracellular signaling cascades including ITAM (Immunoreceptor Tyrosine-based Activation Motif) phosphorylation on CD3 chains via Src family kinases, particularly Lck. Signal 2 involves costimulation between CD28 on T lymphocytes and B7-1 (CD80) or B7-2 (CD86) on APCs, ensuring full activation and T cell proliferation. Signal 3 derives from cytokines (IL-2, IL-12, interferon-α), supporting clonal expansion and differentiation into effector T cells. These signals collectively activate transcription factors (NFAT, NF-κB, AP-1), inducing gene expression necessary for T lymphocyte proliferation and differentiation [14]. 

#### CD8 + T lymphocyte development and function

CD8⁺ T lymphocytes are central mediators of immune surveillance and adaptive immunity against infections and malignancies. Their differentiation and function are tightly regulated by transcription factors, cytokines, chemokines, integrins, and metabolic cues. CD8⁺ cytotoxic T cells serve as the primary effectors for eliminating pathogen-infected and neoplastic cells, whereas CD4⁺ T cells support CD8⁺ responses and mitigate functional exhaustion [15]. CD8⁺ T cells originate from common lymphoid progenitors in the bone marrow and undergo maturation in the thymus. Thymic chemotactic factors guide immature precursors to the thymus, where T-cell receptor (TCR) and CD molecule expression is induced. During thymic selection, positive selection preserves thymocytes with low-affinity recognition of major histocompatibility complex (MHC) molecules, whereas negative selection eliminates self-reactive cells through apoptosis, thereby maintaining self-tolerance. Recognition of MHC class I promotes CD8⁺ lineage commitment, while MHC class II recognition drives CD4⁺ differentiation, with subsequent maturation regulated by cytokine and stromal signals [16]. 

#### Role of cytokines in T-lymphocyte development, maturation, and differentiation

CD8 + T lymphocytes interact with MHC-1 molecules on antigen-presenting cells (APCs) and target cells presenting proteasome-degraded peptide fragments. Direct cell-cell contact and mechanical membrane movement convert mechanical energy into biomechanical signals essential for T-cell receptor (TCR) complex activation. Following chemokine and integrin gradients, activated CD8 + T cells form immunological synapses between supramolecular activation complexes and adhesion molecules on target surfaces. TCR and CD8 co-receptor binding confirm target identity before costimulatory CD28 signals trigger cytotoxic mechanisms [17]. The T-cell receptor complex is composed of an antigen-recognizing TCRαβ heterodimer that is linked through non-covalent interactions to multiple CD3 components, including ζζ, CD3δε, and CD3γε. Each CD3 chain carries ITAM sequences in its cytoplasmic region, which play a critical role in enabling proper receptor assembly inside the cell, its expression on the cell surface, and the transmission of activation signals.TCRα and TCRβ chains each contain variable domains determining antigen specificity, constant domains, membrane-linking peptides, transmembrane regions, and short cytoplasmic tails lacking signaling motifs. CD3 complex transduces intracellular signals requiring CD8 for initiation and amplification. CD45, an abundant T lymphocyte surface glycoprotein, acts as a positive TCR signaling regulator through its intracellular tyrosine phosphatase domain dephosphorylating and activating Lck kinase; Lck subsequently phosphorylates CD3 and ζζ chains, inducing downstream signaling [16]. 

CD8 + T lymphocytes continuously move across target cell surfaces. This mechanical force enhances pore formation on target membranes, triggering death through granule release containing granzymes, perforin, cathepsin C, and granulysin, or alternative endocytic entry pathways. Granulysin and perforin form endosomal pores releasing granzymes into cytoplasm. Additionally, Fas ligand (FASL) on CD8 + T lymphocytes binds target Fas receptors, activating death domains (FADD), triggering caspases and endonucleases inducing DNA fragmentation. Target cell destruction occurs within minutes; single CD8 + T lymphocytes serially or simultaneously eliminate multiple targets. However, cancer cells develop defensive mechanisms including MHC downregulation and perforin-degrading enzymes secretion. Conversely, excessive CD8 + T cell responses cause tissue damage and autoimmunity. To maintain self-tolerance and prevent uncontrolled T lymphocyte activation, CD8 + T cells transiently express inhibitory immune receptors (immune checkpoint molecules) precisely regulating immune responses. Malignant tumors exploit these pathways creating immunosuppressive states supporting survival. Continuous neoantigen exposure induces sustained immune checkpoint expression characterizing T cell exhaustion—a dysfunctional state where antigen elimination alone cannot restore function. Weeks of antigen exposure cause sustained cytotoxic T-lymphocyte-associated protein-4 (CTLA-4) upregulation, ultimately rendering CD8 + T lymphocytes inactive (exhausted) and apoptotic [16]. 

Cytokines are indispensable regulators of T-lymphocyte development, maturation, differentiation, and long-term maintenance throughout adaptive immune responses. During thymic development, interleukin-7 (IL-7) serves as the principal survival cytokine by promoting thymocyte proliferation, T-cell receptor (TCR) gene rearrangement, and prevention of apoptosis through activation of the JAK/STAT5 signaling pathway. IL-7 also supports the survival of naïve T cells after their migration into peripheral lymphoid tissues, thereby maintaining immune homeostasis. Deficiency of IL-7 signaling results in profound impairment of T-cell development and severe lymphopenia. Following antigen recognition, interleukin-2 (IL-2) functions as the major autocrine growth factor for activated T lymphocytes. IL-2 induces rapid clonal expansion, enhances cytotoxic activity, promotes memory T-cell formation, and contributes to the maintenance of immune tolerance by supporting regulatory T-cell (Treg) homeostasis. Additional common γ-chain cytokines, including IL-15 and IL-21, further regulate T-cell persistence and functional differentiation. IL-15 is particularly important for the survival of memory CD8⁺ T cells and natural killer (NK) cells, whereas IL-21 enhances cytotoxicity, cytokine production, and sustained antitumor activity while reducing terminal T-cell exhaustion [16]. 

The differentiation of naïve CD4⁺ T cells into specialized helper subsets is orchestrated by distinct cytokine milieus. IL-12 and interferon-γ (IFN-γ) promote T helper 1 (Th1) polarization, leading to enhanced cellular immunity and activation of macrophages and cytotoxic CD8⁺ T cells. Conversely, IL-4 induces differentiation toward the Th2 lineage, which primarily supports humoral immunity and antibody production. The combination of transforming growth factor-β (TGF-β) with IL-6 promotes differentiation into Th17 cells involved in inflammatory responses, whereas TGF-β together with IL-2 favors the development and maintenance of regulatory T cells that suppress excessive immune activation and preserve self-tolerance. Within the tumor microenvironment, cytokines critically influence T-cell function and therapeutic responsiveness. Immunostimulatory cytokines, including IL-2, IL-12, IL-15, IL-18, and IFN-γ, enhance T-cell proliferation, persistence, and cytotoxic activity against malignant cells. In contrast, immunosuppressive cytokines such as TGF-β and IL-10 impair T-cell activation, promote T-cell exhaustion, and facilitate immune escape by cancer cells. These biological properties have provided the rationale for engineering next-generation CAR-T cells capable of expressing cytokines such as IL-7, IL-12, IL-15, or IL-18, thereby improving persistence, resistance to immunosuppression, and antitumor efficacy in solid tumors, including high-grade serous ovarian carcinoma [16].

#### T lymphocyte roles in antitumor immunity

**Fig. 1:**
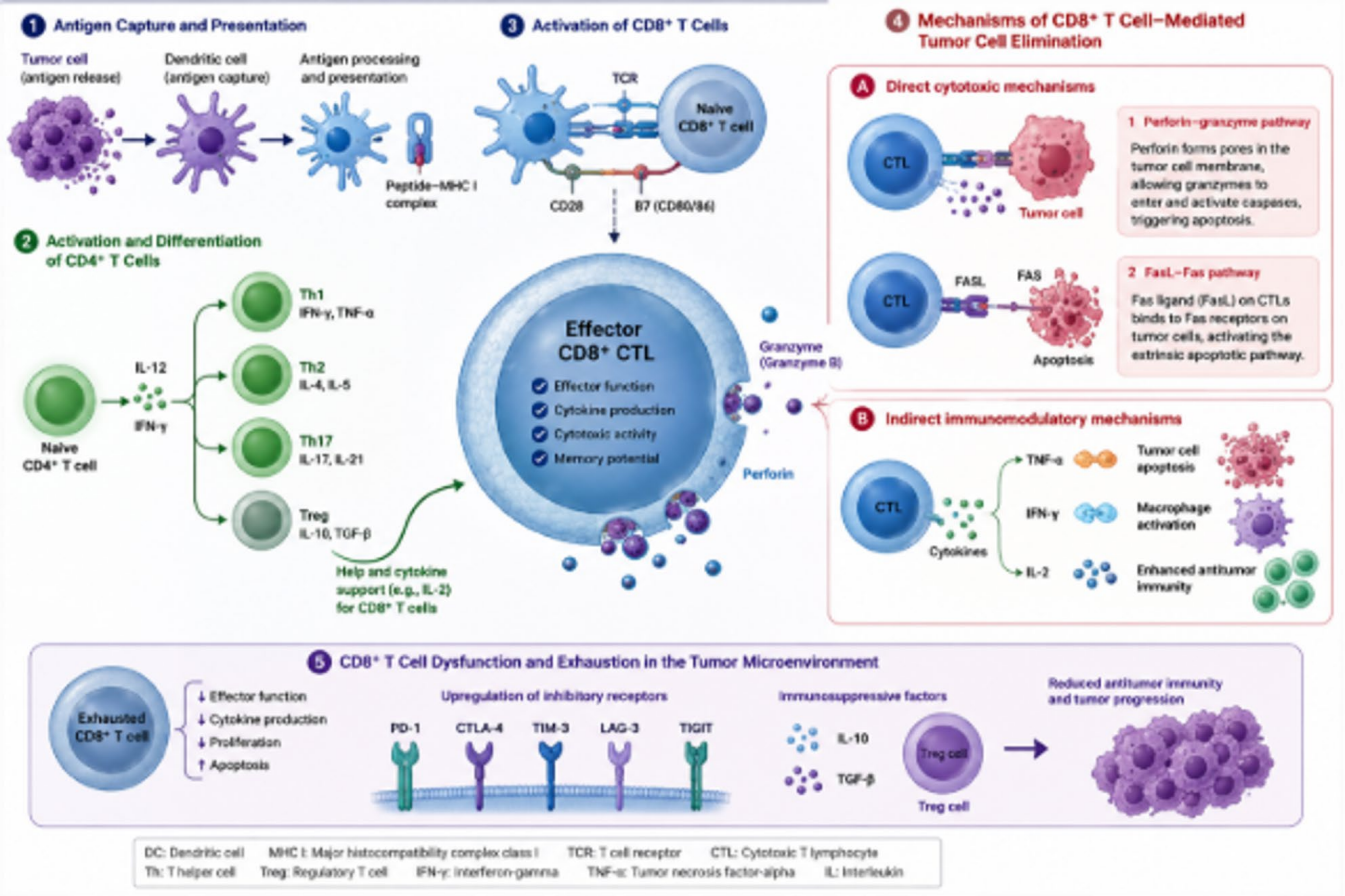
Mechanisms of T-cell–mediated antitumor immunity and CD8⁺ T-cell exhaustion within the tumor microenvironment. This schematic illustration was conceptually designed by the authors and generated with the assistance of artificial intelligence (AI)-based image generation tools, followed by author review and scientific refinement. “This ilustration was Adapted from: Ahmed, H. (2022) ‘Role of T cells in cancer immunotherapy: Opportunities and challenges’, Cancer Pathogenesis and Therapy, 1(2), pp. 116–126. 10.1016/j.cpt.2022.12.002” [21]

Dendritic cells (DCs) continuously survey the tumor microenvironment, capture tumor-associated antigens, and present peptide–major histocompatibility complex (MHC) molecules to naïve T cells within secondary lymphoid organs, thereby initiating adaptive antitumor immunity. Upon activation, CD4⁺ T cells differentiate into specialized subsets, including Th1, Th2, Th9, Th17, and regulatory T (Treg) cells, each characterized by distinct cytokine profiles and immunological functions. CD4⁺ T cells play a pivotal role in antitumor responses by priming cytotoxic CD8⁺ T cells, sustaining effector-memory function, and directly mediating tumor cell elimination. Furthermore, CD4⁺ T cell-derived interferon-γ (IFN-γ) enhances antitumor immunity by inducing anti-angiogenic chemokines, activating macrophages, and promoting tumoricidal effector mechanisms. Effective tumor control, however, depends on the coordinated interaction between CD4⁺ T cells, IFN-γ, and other immune components within the tumor microenvironment [18–21]. CD8⁺ cytotoxic T lymphocytes (CTLs) are principal effector cells in antitumor immunity and cancer immunotherapy. Their activation and differentiation require coordinated interactions among antigen-presenting cells (APCs), CD4⁺ helper T cells, and cytokine-mediated signaling, particularly interferon-γ (IFN-γ). Upon maturation, effector CD8⁺ T cells acquire potent cytotoxic activity and mediate tumor eradication through both direct cytolytic mechanisms and indirect immunomodulatory pathways. Mechanisms of CD8⁺ T Cell–Mediated Tumor Cell Elimination (Fig. 1). CD8⁺ cytotoxic T lymphocytes (CTLs) mediate tumor cell elimination through both direct and indirect mechanisms. Direct cytotoxicity is primarily achieved via perforin–granzyme release following immune synapse formation, whereby perforin facilitates granzyme entry into target cells, triggering apoptotic cell death. CTLs can also induce apoptosis through Fas ligand (FasL) interaction with Fas receptors expressed on tumor cells, activating the extrinsic caspase-dependent pathway. In addition to these contact-dependent mechanisms, CTLs secrete cytokines, particularly tumor necrosis factor-α (TNF-α), which promote tumor cell apoptosis and enhance antitumor immunity independently of direct cellular interaction. Both CD4⁺ and CD8⁺ T cells generate memory populations that provide long-term immunological protection; however, persistent antigen exposure within chronic infections and the tumor microenvironment can drive T-cell exhaustion. This dysfunctional state is characterized by progressive loss of effector function, reduced cytokine production, and sustained expression of inhibitory immune checkpoint receptors, including programmed cell death protein-1 (PD-1), cytotoxic T-lymphocyte-associated protein-4 (CTLA-4), T-cell immunoglobulin and mucin-domain containing-3 (TIM-3), and lymphocyte-activation gene-3 (LAG-3), ultimately impairing antitumor immunity and facilitating immune evasion [21]. 

##### Perforin/granzyme-dependent direct cytotoxicity

The classical mechanism of CTL-mediated tumor cell killing requires intimate intercellular contact between the effector lymphocyte and target cell. Upon immune synapse formation, CTLs release cytotoxic granules containing the pore-forming protein perforin and serine protease granzyme B. Perforin oligomerizes within the target cell membrane, creating membrane disruptions that facilitate granzyme B internalization. Once cytoplasmic entry is achieved, granzyme B cleaves key cellular substrates and activates pro-apoptotic caspases, ultimately triggering programmed cell death in the targeted neoplastic cell [21]. 

##### Fas ligand-mediated direct apoptosis

An alternative direct killing mechanism operates through death receptor ligation. CTLs constitutively express Fas ligand (Fas-L), which engages the Fas death receptor frequently upregulated on malignant cells. This receptor-ligand interaction recruits adaptor proteins and initiates caspase-8 activation through the extrinsic apoptotic pathway, resulting in caspase-dependent programmed cell death of Fas-expressing tumor cells [21]. 

##### Cytokine-mediated indirect anti-tumor effects

Beyond direct cytolytic mechanisms, CTLs exert anti-tumor effects through paracrine and systemic cytokine secretion. Tumor necrosis factor-alpha (TNF-α) released by CTLs acts at a distance on TNF receptor-expressing tumor cells, triggering apoptotic cascades independent of direct cellular contact. This “bystander” killing mechanism enables CTLs to influence tumor cell fate across microenvironmental distances, potentially targeting cells beyond the immediate immunological synapse [21]. 

#### T cell exhaustion in cancer: mechanisms and therapeutic recovery

**Fig. 2:**
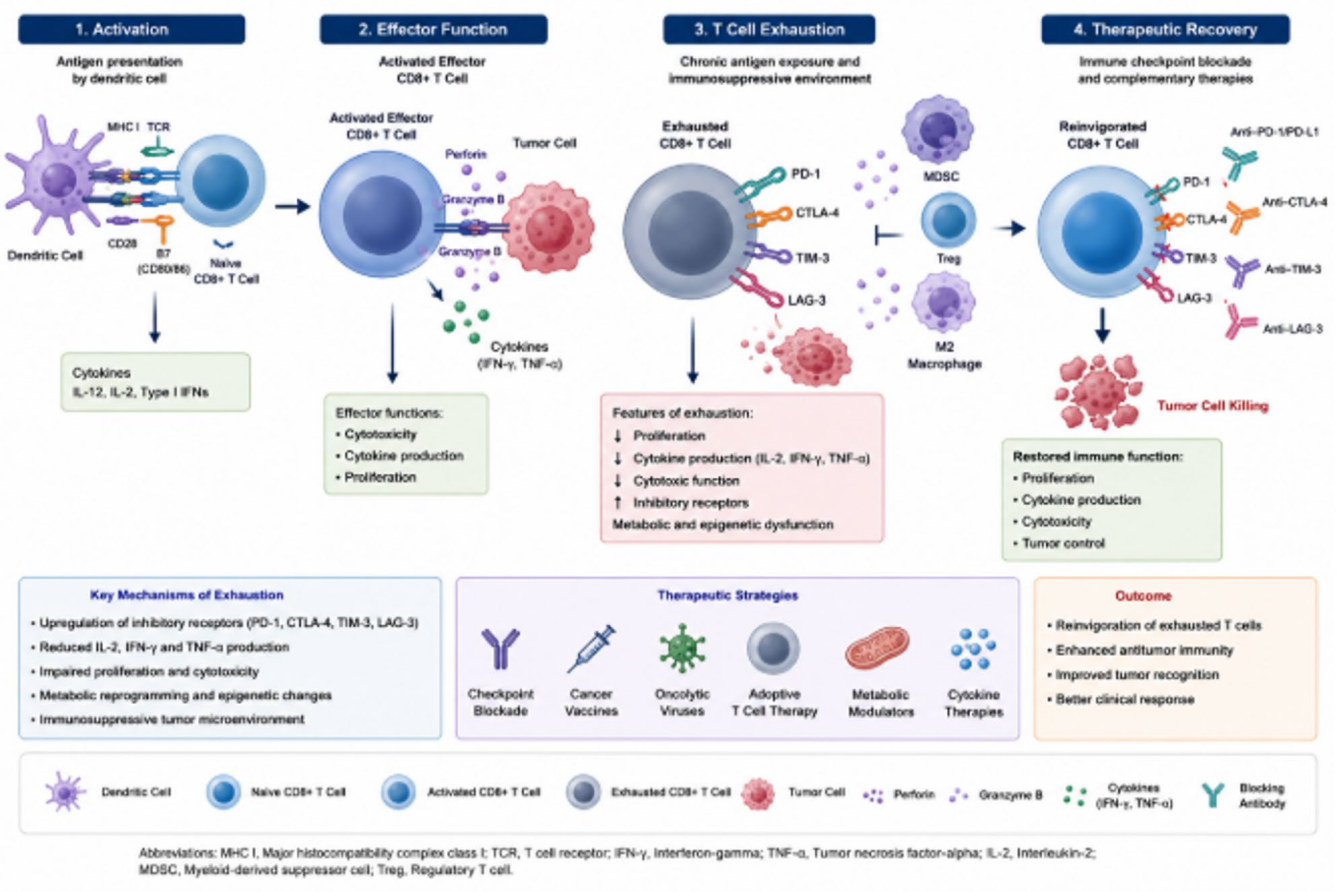
T-cell Exhaustion in the Tumor Microenvironment: Mechanisms and Therapeutic Recovery. This schematic illustration was conceptually designed by the authors and generated with the assistance of artificial intelligence (AI)-based image generation tools, followed by author review and scientific refinement. “This ilustration was Adapted from: Ahmed, H. (2022) ‘Role of T cells in cancer immunotherapy: Opportunities and challenges’, Cancer Pathogenesis and Therapy, 1(2), pp. 116–126. 10.1016/j.cpt.2022.12.002” [21]

T-cell exhaustion is a progressive dysfunctional state characterized by sustained expression of inhibitory immune checkpoint receptors and impaired effector function within the tumor microenvironment. One of the earliest and most critical events in this process is the upregulation of programmed cell death protein−1 (PD−1), which, upon interaction with its ligands PD-L1 and PD-L2 on tumor or antigen-presenting cells, transmits inhibitory signals that suppress T-cell proliferation, cytokine production, and cytotoxic activity. Cytotoxic T-lymphocyte-associated protein−4 (CTLA−4) further contributes to T-cell dysfunction through its interaction with CD80/CD86, resulting in reduced T-cell activation, diminished interleukin−2 (IL−2) production, and weakened antitumor immunity. In addition, exhausted T cells frequently co-express other inhibitory receptors, including T-cell immunoglobulin and mucin-domain containing−3 (TIM−3) and lymphocyte-activation gene−3 (LAG−3), which provide complementary suppressive signals that reinforce functional impairment. Despite these profound alterations, T-cell exhaustion remains partially reversible. Immune checkpoint blockade using anti-PD−1 and anti-CTLA−4 monoclonal antibodies can restore effector function, enhance cytokine production, and improve tumor cell recognition. Furthermore, therapeutic vaccines and other immunomodulatory strategies can synergistically reinvigorate exhausted T cells, thereby promoting more effective and sustained antitumor immune responses (Fig. 2) [21]. 

##### PD−1 checkpoint-mediated exhaustion

Upregulation of PD−1 on exhausted T cells represents an early critical event in T cell dysfunction. PD−1 engagement with its ligands (PD-L1/PD-L2) on tumor or antigen-presenting cells transmits inhibitory signals that suppress T cell proliferation, cytokine production, and effector function, contributing substantially to T cell exhaustion [21]. 

##### CTLA−4-mediated exhaustion

CTLA−4 expression on the T cell surface constitutes another major immune checkpoint driving exhaustion. CTLA−4 engagement with CD80/CD86 molecules generates potent inhibitory signals that further reduce T cell activation, IL−2 production, and anti-tumor immunity, exacerbating the exhausted phenotype [21]. 

##### Additional checkpoint receptors (TIM−3 and LAG−3)

Exhausted T cells co-express additional inhibitory receptors TIM−3 and LAG−3, which engage their respective ligands and provide cumulative suppressive signals. This multilayered checkpoint blockade consolidates T cell exhaustion and functional impairment [21]. The exhausted T cell state, though profound, remains therapeutically reversible. Anti-PD−1 and anti-CTLA−4 monoclonal antibodies block inhibitory signaling and restore partial T cell functionality. Therapeutic vaccines enhance tumor-specific T cell responses, while complementary immunotherapies collectively reinvigorate exhausted T lymphocytes, restore cytokine production, and enable effective tumor cell recognition and elimination [21]. 

### Engineered T lymphocyte technologies: CAR-T and TCR-T cell design and mechanisms

#### Engineered T lymphocyte development

T lymphocyte engineering core principles center on gene transduction determining and enhancing CD8 + T cell functionality. Initial approaches employed retroviral vectors cloning tumor-specific TCRα and β chains, generating CD8 + T lymphocytes MHC-I-dependently recognizing tumor-associated antigens. However, this process proved complex and expensive. To eliminate autologous tumor-reactive CD8 + T cell isolation and expansion requirements, current CAR T-cell production uses bulk peripheral blood T lymphocytes, revolutionizing adoptive cell transfer in oncology [17, 22, 23]. CD8 + cytotoxic T lymphocytes from adaptive immunity represent the most potent effectors of antitumor responses and form the backbone of cancer immunotherapy. Immune checkpoint inhibitors revive dysfunctional T lymphocytes by blocking suppressive receptors, while adoptive cell transfer deploys genetically modified CD8 + T cells with engineered receptors (chimeric antigen receptors [CARs]). Both approaches have revolutionized cancer treatment efficacy and personalized therapeutic options; however, durable responses remain inconsistent and adverse effects frequently necessitate treatment cessation, highlighting the need for continuous strategy refinement [24]. 

#### CAR T-cell therapy

CAR-T cell therapy represents a revolutionary approach that empowers a patient’s own T cells to fight cancer by introducing a synthetic receptor that combines the antigen-binding specificity of monoclonal antibodies with the cytotoxic machinery of T lymphocytes [25–27]. CAR T-cell production involves synthetic CAR construction through single-chain variable fragment (scFv) immunoglobulin fusion with intracellular signaling domains, typically including transmembrane domains and CD3-ζ endodomains, followed by autologous T lymphocyte expansion and patient reinfusion. CARs recognize any tumor cell surface structure (protein, carbohydrate, glycolipid) independently from APCs and MHC presentation [17, 22, 23]. CAR molecules’ extracellular portions typically derive from monoclonal antibodies against target antigens. Heavy variable (VH) and light variable (VL) chains (single-chain variable fragments [scfv]) link via linkers forming CAR antigen-specific components. Hinge/spacers connect scfv to transmembrane domains piercing cell membranes. Intracellular domains contain co-stimulatory components and CD3ζ chains transmitting signals after scfv CAR recognition and tumor antigen binding. Co-stimulatory signaling depends on domain type: CD28 requires PI3K activation while 4-1BB requires tumor necrosis factor receptor-associated factors (TRAFs) and NF-κB activation. CD3ζ contains three immunoreceptor tyrosine-based activation motifs (ITAMs) undergoing phosphorylation triggering ζ-associated protein of 70 kDa (ZAP70) signals. Downstream signaling activates CD8 + T lymphocyte effector functions including perforin and granzyme release causing tumor cell death. T lymphocyte activation additionally produces IL-2 supporting T cell proliferation and activity [28]. 

The evolution of CAR technology has progressed through multiple generations, each incorporating structural refinements to enhance therapeutic efficacy. First-generation CARs consisted of a single-chain variable fragment (scFv) targeting a tumor-associated antigen fused to the CD3ζ signaling domain but demonstrated limited persistence and expansion in vivo due to inadequate costimulation [25–27]. First-generation CARs contained scFv-linked antigen-binding through protein hinges/spacers to transmembrane and CD3ζ intracellular domains. Hinges determine target accessibility; extended hinges provide flexibility for membrane-proximal antigens or complex epitopes [17, 22, 23]. Second-generation CARs, which incorporated costimulatory domains such as CD28 or 4-1BB alongside CD3ζ, markedly improved CAR-T cell proliferation, cytokine production, and persistence, leading to the current FDA-approved products for hematologic malignancies. Third-generation CARs integrated multiple costimulatory domains to further enhance T cell activation, although clinical data comparing second and third-generation constructs remain inconsistent [25–27]. Second and third-generation CARs insert co-stimulatory signaling domains (typically CD28 or CD137/4-1BB) between transmembrane and ζ chain domains, addressing first-generation T-cell anergy and supporting expansion and persistence [17, 22, 23]. Fourth-generation CARs, also termed TRUCKs (T cells redirected for antigen-unrestricted cytokine-initiated killing), are engineered to secrete transgenic cytokines such as IL-12, IL-7, IL-15, or IL-18 upon CAR engagement, thereby modulating the immunosuppressive TME and recruiting endogenous immune cells. Fifth-generation CARs incorporate universal platforms including SUPRA CAR and BBIR CAR systems that offer enhanced controllability and adaptability [25–27]. Fourth and fifth-generation CARs incorporate cytokine receptor signaling domains (IL-12, IL-18), further enhancing T lymphocyte populations without systemic interleukin toxicity [17, 22, 23]. 

#### TCR-T cell therapy

**Fig. 3:**
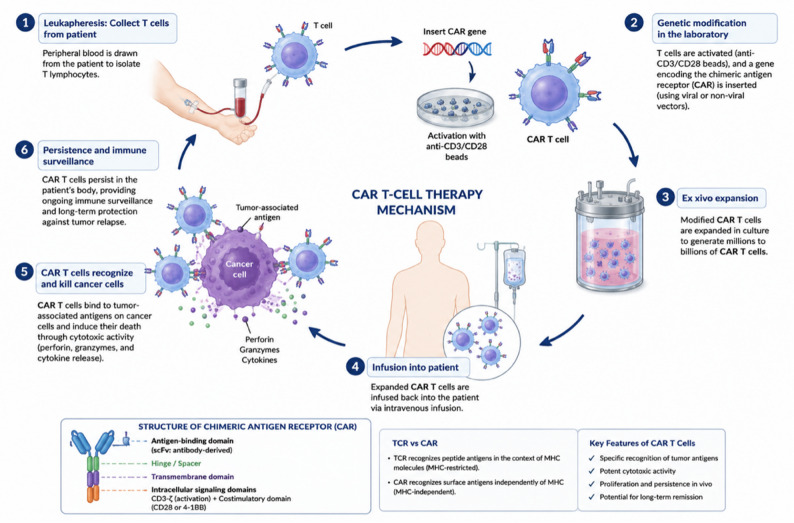
Workflow and mechanism of CAR-T cell therapy in cancer. This schematic illustration was conceptually designed by the authors and generated with the assistance of artificial intelligence (AI)-based image generation tools, followed by author review and scientific refinement. Adapted from: Zhao, Q. (2021) ‘Engineered TCR-T cell immunotherapy in anticancer precision medicine: Pros and cons’, Frontiers in Immunology, 12, p. 658,753. 10.3389/fimmu.2021.658753

Beyond CAR-T, TCR-T represents another engineered T lymphocyte approach. Both TCR-T and CAR-T are genetically modified peripheral blood cells recognizing tumor antigens and enhancing adoptive cell therapy (ACT) efficacy. Pre-reinfusion requires activation (anti-CD3/CD28 beads) and expansion. TCR and CAR possess distinct molecular structures recognizing different antigens. TCRs comprise α and β chains recognizing processed, MHC-presented peptides; transgenic TCRs target diverse tumor antigens as both intracellular and surface proteins present as peptides in MHC contexts. CARs are artificial receptors comprising antibody heavy and light variable regions linked to intracellular signaling chains (CD3-ζ, CD28, 4-1BB). CAR-recognized antigens need not be MHC-restricted but must express on tumor surfaces (Fig. 3) [28]. 

#### CD4 + T cell-based immunotherapy strategy in cancer treatment

**Fig. 4:**
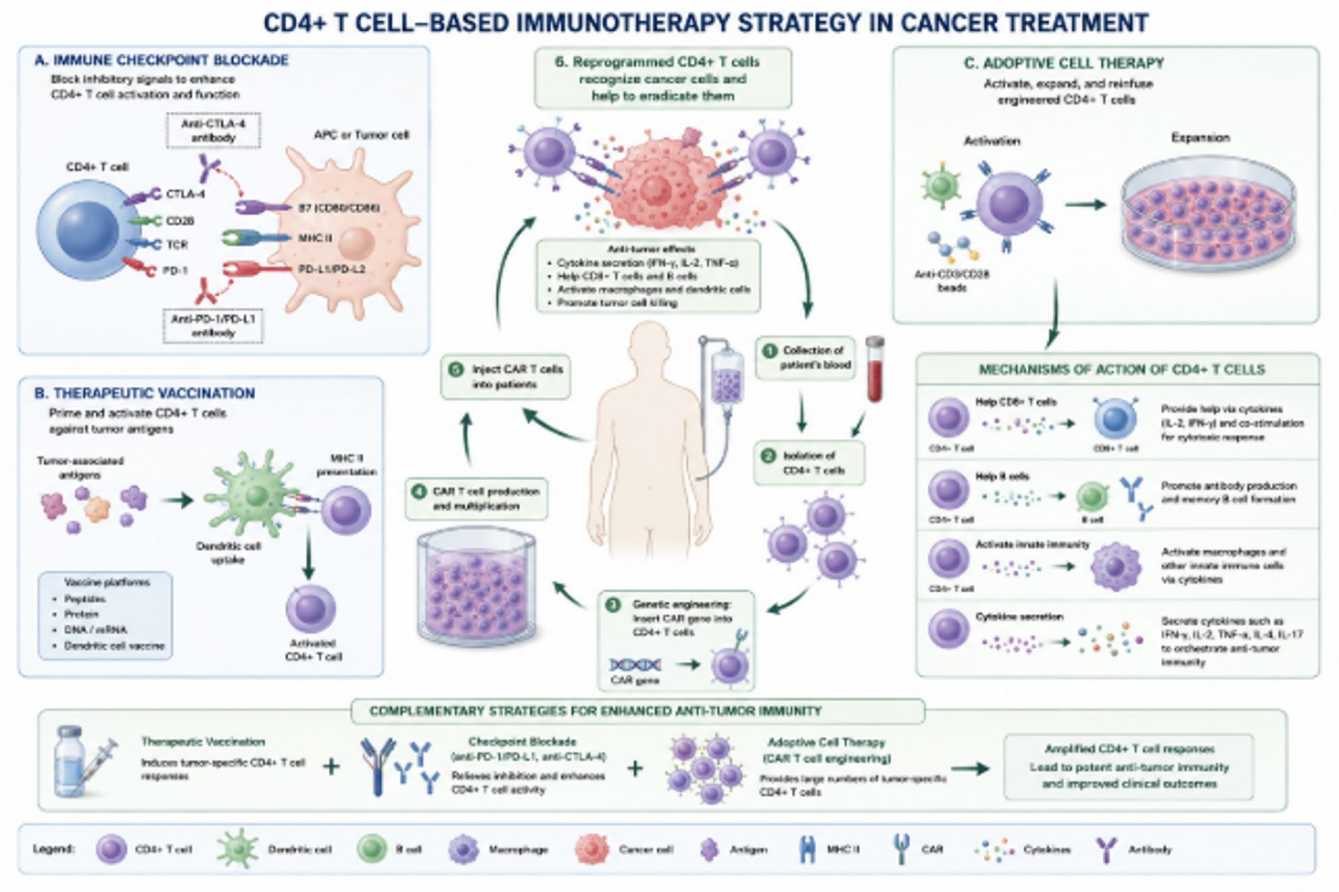
CD4 + T Cell-Based Immunotherapy Strategy in Cancer Treatment. This schematic illustration was conceptually designed by the authors and generated with the assistance of artificial intelligence (AI)-based image generation tools, followed by author review and scientific refinement. “This ilustration was Adapted from: Ahmed, H. (2022) ‘Role of T cells in cancer immunotherapy: Opportunities and challenges’, Cancer Pathogenesis and Therapy, 1(2), pp. 116–126. 10.1016/j.cpt.2022.12.002” [21]

CD4 + T cells play a pivotal role in cancer immunotherapy; however, cancer cells frequently evade immune surveillance through expression of immune checkpoint molecules. To overcome this immune evasion, CD4 + T cells can be therapeutically primed using either generalized or personalized vaccination strategies tailored to individual patient white blood cell profiles and tumor-specific antigens. Patient blood is collected and subjected to ex vivo reprogramming via two complementary approaches: adoptive cell therapy and chimeric antigen receptor (CAR) T cell engineering. In CAR T cell therapy, CAR genes are introduced into autologous CD4 + T cells and amplified to achieve therapeutic cell numbers. These genetically modified CAR T cells are subsequently reinfused into the patient’s circulation, where they execute targeted cytotoxic attacks against cancer cells expressing cognate antigens. In adoptive cell therapy, engineered CD4 + T cell populations are activated through engagement with tumor-associated antigens or professional antigen-presenting cells, thereby amplifying the overall anti-tumor immune response. Collectively, these complementary immunotherapeutic strategies—therapeutic vaccination, CAR T cell engineering, and adoptive cell therapy—represent potent approaches to harness CD4 + T cell effector functions for cancer treatment (Fig. 4) [21].

### Engineered T lymphocytes in ovarian cancer and high grade serous ovarian carcinoma

Despite significant advances in surgical techniques and chemotherapeutic regimens over the past decades, ovarian cancer remains the most lethal gynecologic malignancy worldwide, with high-grade serous ovarian carcinoma (HGSOC) accounting for approximately 75% of cases and most deaths [29–32]. 

This dire prognosis has catalyzed intense research into novel therapeutic modalities, with engineered T lymphocyte-based immunotherapy emerging as a promising frontier in the treatment landscape. The rationale for immunotherapeutic approaches is compelling: HGSOC tumors demonstrate inherent immunogenicity, evidenced by the presence of tumor-infiltrating lymphocytes (TILs) that correlate with improved survival outcomes, and the detection of tumor antigen-reactive T cells and antibodies in patient blood and ascites. However, the therapeutic efficacy of naturally occurring antitumor T cells is constrained by multiple immunosuppressive mechanisms within the tumor microenvironment (TME), suboptimal T cell avidity, and the limited availability of tumor-reactive lymphocytes. Engineered T cell therapy represents a transformative strategy to overcome these limitations by genetically modifying patient-derived T cells to express high-affinity tumor-targeting receptors, thereby redirecting the immune system to mount a robust and sustained attack against cancer cells [29–32]. 

#### Molecular and immunological landscape of high-grade serous ovarian carcinoma genomic characterization and molecular heterogeneity

High-grade serous ovarian carcinoma (HGSOC) is a molecularly heterogeneous malignancy characterized by extensive chromosomal instability, recurrent copy number alterations, and near-universal TP53 mutations. Frequent genomic aberrations include losses of NF1, RB1, and PTEN, as well as amplifications of CCNE1 and MYC, contributing to substantial inter- and intratumoral heterogeneity. Approximately half of HGSOC tumors exhibit homologous recombination (HR) deficiency, most commonly due to BRCA1/BRCA2 alterations, which confer sensitivity to poly(ADP-ribose) polymerase (PARP) inhibitors through synthetic lethality. Transcriptomic analyses further classify HGSOC into distinct molecular subtypes, including immunoreactive, mesenchymal, proliferative, and differentiated phenotypes. Among these, the immunoreactive subtype is characterized by increased immune-cell infiltration and enhanced expression of antigen-presentation and T-cell activation pathways, features associated with improved clinical outcomes and greater responsiveness to immunotherapeutic strategies [32–34]. The tumor microenvironment (TME) of HGSOC constitutes a major barrier to effective antitumor immunity by establishing a profoundly immunosuppressive milieu. This environment is characterized by the accumulation of regulatory T cells (Tregs), tumor-associated macrophages (TAMs), and myeloid-derived suppressor cells (MDSCs), which collectively suppress effector T-cell function through the production of immunosuppressive cytokines such as interleukin-10 (IL-10) and transforming growth factor-β (TGF-β), as well as the expression of inhibitory immune checkpoint molecules. HGSOC cells further promote immune evasion through the expression of immune checkpoint ligands, including programmed death-ligand 1 (PD-L1), leading to T-cell dysfunction and exhaustion. In addition, metabolic competition within the TME deprives T cells of essential nutrients while generating inhibitory metabolites that impair their activation and persistence. Dense stromal architecture and abnormal tumor vasculature further restrict immune-cell infiltration. Collectively, these mechanisms limit effective antitumor immune responses and contribute to therapeutic resistance in HGSOC [25–27, 30]. 

#### Chimeric antigen receptor T cell CAR-T) therapy in HGSOC

##### Target antigens in ovarian cancer CAR-T therapy

The identification of suitable target antigens represents a critical challenge in developing CAR-T therapies for HGSOC, as ideal targets must be highly expressed on tumor cells while demonstrating minimal expression in normal tissues to avoid on-target, off-tumor toxicity. Several candidate antigens have been extensively investigated: [25–27, 32–34].

##### Mesothelin (MSLN)

Emerges as one of the most promising targets, being overexpressed in more than 75% of HGSOC tumors while exhibiting limited expression in healthy mesothelial cells lining the pleura, peritoneum, and pericardium. MSLN contributes to the malignant and invasive phenotype of ovarian cancer, and its tumor-promoting functions make antigen loss disadvantageous for cancer cells. Multiple preclinical studies have demonstrated that MSLN-targeting CAR-T cells can effectively infiltrate ovarian tumors and exert cytotoxic activity against MSLN-positive cancer cells. Several early-phase clinical trials have evaluated anti-MSLN CAR-T therapy in ovarian cancer patients. A University of Pennsylvania phase I trial examined fully human M5 CAR-T cells incorporating a 4−1BB costimulatory domain in patients with mesothelin-expressing tumors. Preliminary data demonstrated that M5 CAR-T cells expanded in patient blood with peak levels at days 7–10, and expansion was 10-fold higher when preceded by lymphodepletion. The treatment achieved disease control in a portion of patients (8/14) within about one month post-administration, but failed to produce measurable tumor regression such as complete or partial responses. Higher dosing levels were associated with notable toxicities, including grade 3 CRS and respiratory adverse effects. Findings from Chen et al. indicate that autologous anti-MSLN CAR-T cells can provide temporary disease control in ovarian cancer, yielding progression-free intervals of roughly 4 to 6 months, without significant neurologic events or severe CRS. In a separate investigator-led study, intraperitoneal delivery of CAR-T cells engineered to release IL−7 and CCL19 was safe but ultimately ineffective in halting disease progression. More recently, a novel DAP-CAR-T platform targeting MSLN has demonstrated improved therapeutic potential, with early clinical data showing partial tumor responses in some patients and disease stabilization in others, accompanied by a favorable tolerability profile [25–27, 32–34]. 

##### Folate receptor alpha (FRα)

Represents another extensively studied target, expressed in approximately 80–90% of ovarian carcinomas while demonstrating limited physiologic expression on the apical surfaces of polarized epithelial cells in kidneys, lungs, and intestinal tissues where it remains largely inaccessible to circulating antibodies or CAR-T cells. An ongoing first-in-human phase I clinical trial (NCT01567891) is evaluating FRα-directed CAR-T cells with dual 4−1BB and TCRζ signaling domains, utilizing a MOv19 anti-FRα single-chain variable fragment. Patients receive intraperitoneal administration of CAR-T cells with or without antecedent lymphodepleting chemotherapy in women with recurrent high-grade serous ovarian cancer expressing ≥ 2+ FRα staining in ≥ 70% of tumor cells. While complete results are pending, preliminary data have not shown tumor burden reduction, suggesting that additional strategies to overcome immunosuppression may be required. A recent phase I trial evaluating FSHR-targeting CAR-T cells in ovarian cancer has successfully dosed patients in three cohorts with dose escalation, advancing to a 10-fold higher dose without dose-limiting toxicities, with one patient demonstrating relative stability and mild improvement for over one year after infusion [25–27, 32–34]. 

##### MUC16 (CA125)

Highly relevant in ovarian cancer as the standard diagnostic marker and is overexpressed in more than 80% of epithelial ovarian cancers. While the cleaved extracellular domain (CA125) circulates in serum, the membrane-associated repeat domains represent attractive CAR targets. Recent studies have validated that the CA125 extracellular repeat domain of MUC16 can be effectively targeted by CAR-T cells. The K101CAR construct demonstrated high efficacy against cell lines and patient-derived tumors in vitro and in vivo, with functionality not impaired by soluble CA125 antigen. A phase I clinical trial (NCT06469281) is currently evaluating 27T51, a CAR-T therapy targeting MUC16, in combination with checkpoint blockade and anti-angiogenic therapy at multiple centers including Roswell Park Comprehensive Cancer Center. Earlier clinical investigation of MUC16ecto-targeting CAR-T cells engineered to secrete IL−12 demonstrated feasibility of intraperitoneal administration, though clinical responses remained limited [25–27, 32–34]. 

##### B7-H3 (CD276)

Emerged as a promising target highly expressed in ovarian cancers and many other solid tumors while demonstrating restricted expression in normal tissues. Multiple phase I clinical trials are evaluating B7-H3-directed CAR-T cell therapy in ovarian cancer. A Stanford-led trial is investigating B7-H3-targeting CAR-T cells administered both intravenously and via direct intraperitoneal injection; preliminary findings from the first six treated patients show encouraging initial responses with manageable side effects. Another ongoing phase I study (NCT06305299) is testing autologous iC9-CAR.B7-H3 T cells incorporating an inducible caspase−9 safety switch in patients with platinum-resistant epithelial ovarian cancer, with intraperitoneal administration and dose escalation to determine the maximum tolerated dose and recommended phase 2 dose. Preclinical studies have demonstrated that B7-H3-directed CAR-T cells can effectively target and eliminate B7-H3-positive ovarian cancer cells while recruiting endogenous immune responses [25–27, 32–34]. 

Other Targets under investigation include HER2, which is overexpressed in subsets of ovarian cancers; SSEA−4, a glycosphingolipid antigen that demonstrated remarkable antitumor responses in high-grade serous ovarian cancer xenograft models, though safety concerns at high CAR-T doses necessitate dose-limiting or combinatorial targeting strategies; and Tag72, which has been evaluated in early CAR-T clinical trials [25–27, 32–34]. 

#### Clinical challenges and limitations of CAR-T therapy in HGSOC

Despite promising preclinical data, CAR-T cell therapy has achieved only modest clinical success in ovarian cancer to date, with objective response rates typically ranging from 8 to 15% as monotherapy and up to 31% with combination approaches, far below the dramatic remission rates observed in hematologic malignancies. Multiple interconnected factors contribute to this therapeutic resistance: Tumor Antigen Heterogeneity and Escape: HGSOC exhibits significant inter- and intra-tumoral heterogeneity in antigen expression, enabling tumor subpopulations lacking the target antigen to escape CAR-T-mediated cytotoxicity. This antigen escape represents a major mechanism of relapse following CAR-T therapy, as cancer cells may downregulate or lose target antigen expression under immune pressure. Strategies to address this challenge include dual-antigen or tandem CAR approaches that simultaneously target multiple antigens, reducing the likelihood of complete immune evasion [25–27, 32–34]. 

There are four major categories of obstacles that limit CAR-T cell efficacy in ovarian cancer:

A. Physical Barriers to T Cell Trafficking: The peritoneal dissemination pattern of ovarian cancer, dense stromal architecture, and aberrant tumor vasculature limit CAR-T cell infiltration into tumor deposits. While intraperitoneal administration of CAR-T cells offers the potential advantage of circumventing systemic trafficking barriers by delivering cells directly to the tumor-bearing compartment, this approach requires specialized catheter placement and may be associated with localized toxicities [25–27, 32–34].
B. Immunosuppressive Tumor Microenvironment: The hostile ovarian cancer TME, characterized by accumulation of Tregs, myeloid-derived suppressor cells, and tumor-associated macrophages along with secretion of immunosuppressive cytokines and expression of inhibitory ligands, actively suppresses CAR-T cell function. This immunosuppression induces progressive T cell exhaustion, characterized by upregulation of multiple inhibitory receptors (PD−1, TIM−3, LAG−3, TIGIT), decreased effector cytokine production, impaired proliferative capacity, and ultimately physical deletion of severely exhausted T cells [25–27, 32–34].
C. Limited Persistence and T Cell Exhaustion: CAR-T cells in ovarian cancer models demonstrate initial tumor infiltration but progressively lose function over time, becoming exhausted within 21–28 days of transfer. This exhaustion results from persistent antigen stimulation, engagement of inhibitory receptors, metabolic stress, and exposure to immunosuppressive signals within the TME. Studies have shown that while tumor-specific CAR-T cells preferentially accumulate in ovarian tumors compared to non-specific T cells, they exhibit limited Ki67 expression indicating poor proliferation within tumor sites and undergo progressive numerical decline [25–27, 32–34].
D. Toxicities: CAR-T therapy carries risks of serious adverse events including cytokine release syndrome (CRS), hemophagocytic lymphohistiocytosis/macrophage activation-like syndrome (HLH/MAS), and neurotoxicity. While these toxicities have been less prominent in ovarian cancer trials compared to hematologic malignancy trials, likely reflecting lower tumor burdens and reduced CAR-T expansion, dose-limiting toxicities have been observed, particularly respiratory complications in patients with high pulmonary tumor burden. The incorporation of safety switches such as inducible caspase−9 into CAR constructs represents an important strategy to mitigate excessive toxicity by enabling conditional elimination of CAR-T cells if severe adverse events occur [25–27, 32–34].

#### T cell receptor-engineered T cell therapy

##### TCR-T cell technology and mechanisms

T cell receptor-engineered T cell (TCR-T) therapy represents an alternative approach to redirect T cells against cancer, wherein patient-derived T cells are genetically modified to express high-affinity TCRs specific for tumor antigens presented by human leukocyte antigen (HLA) molecules. Unlike CAR-T cells that recognize surface antigens in an MHC-independent manner, TCR-T cells can target intracellular proteins that are processed and presented as peptide-MHC complexes on tumor cell surfaces, dramatically expanding the universe of targetable antigens. This MHC-restricted recognition enables TCR-T cells to target driver mutations, neoantigens, and other intracellular tumor-associated proteins that would be inaccessible to CAR-T therapy. The technology has demonstrated remarkable clinical success in certain solid tumors, with NY-ESO-1-targeting TCR-T cells achieving objective response rates of 55% in metastatic melanoma and 61% in synovial sarcoma [35]. 

##### TCR-T therapy targeting mesothelin in HGSOC

Anderson et al. conducted seminal studies evaluating engineered adoptive T cell therapy in HGSOC using CD8 + T cells transduced with high-affinity TCRs specific for mesothelin. Using the ID8VEGF murine ovarian cancer model that recapitulates key immunologic features of human HGSOC including suppressive immune cell infiltration and expression of T cell-inhibitory molecules, the investigators demonstrated that mesothelin-specific TCR-engineered T cells (TCR1045) could preferentially infiltrate established ovarian tumors compared to control T cells (14.31% vs. 3.23% of tumor-infiltrating T cells). Human CD8 + T cells engineered with a mesothelin-specific TCR (TCR530) demonstrated tumoricidal activity against three HLA-A2 + HGSOC cell lines. However, single-dose therapy showed limited efficacy as transferred T cells underwent progressive dysfunction and failed to persist beyond 28 days. To address this limitation, the investigators implemented a combinatorial regimen involving lymphodepletion with cyclophosphamide, repeated doses of TCR-engineered T cells administered every 14 days, peptide-pulsed irradiated splenocyte vaccines, and IL-2 support. This approach significantly prolonged survival of tumor-bearing mice (median survival 112 days versus 77 days for untreated controls), affirming that repeated infusions of functional T cells could maintain therapeutic efficacy despite progressive exhaustion of individual T cell populations. Importantly, tumors from treated mice demonstrated significantly increased cleaved caspase-3 staining indicative of ongoing tumor cell killing, without evidence of toxicity to MSLN-expressing mesothelial surfaces, and retained MSLN expression at late timepoints, suggesting that therapeutic failure resulted from T cell dysfunction rather than antigen loss [30]. 

##### Engineering strategies to overcome TME immunosuppression

Recognizing that FasL-mediated apoptosis represents a significant barrier to T cell persistence in ovarian cancer, with FasL expression detected on tumor vasculature and cancer cells, Anderson et al. developed immunomodulatory fusion proteins (IFPs) that convert death signals into pro-survival signals. These IFPs consist of the Fas extracellular binding domain fused to the 4-1BB costimulatory domain rather than the native death domain. Mesothelin-specific TCR-engineered T cells co-expressing Fas-4-1BB IFPs demonstrated superior persistence in ovarian tumor-bearing mice, with improved proliferation and reduced apoptosis compared to T cells expressing only the TCR. Critically, TCR-engineered T cells with the IFP significantly prolonged mouse survival without inducing on-target, off-tumor toxicity. Human T cells expressing mesothelin-specific TCR530 and Fas-4-1BB IFP exhibited enhanced functional activity and viability compared to cells with TCR alone. This innovative approach demonstrates the potential of engineering T cells to resist specific immunosuppressive mechanisms within the ovarian cancer TME [26]. 

##### Neoantigen-targeting TCR-T therapy

Neoantigens—novel epitopes arising from tumor-specific somatic mutations—represent ideal targets for TCR-T therapy as they are truly tumor-specific, minimizing risks of on-target, off-tumor toxicity while potentially generating highly immunogenic epitopes. Multiple groups have developed efficient pipelines for identifying neoantigen-specific T cells and their cognate TCRs in ovarian cancer patients. Liu et al. demonstrated that using prioritized neoantigen candidates, spontaneous CD4 + and/or CD8 + T cell responses against neoepitopes could be detected in half of treatment-naïve HGSOC patients, with a validation rate of 19%. Importantly, T cells specific for mutated cancer-associated genes including NUP214 and JAK1 recognized and killed autologous tumors, and genetic transfer of TCRs from these neoantigen-specific T cell clones conferred neoantigen reactivity to peripheral blood T cells. Tumors from patients exhibiting neoantigen-specific T cell responses demonstrated upregulation of antigen processing and presentation machinery, which associated with favorable patient survival in the TCGA ovarian cohort. Matsuda et al. validated an efficient protocol for producing neoantigen-specific TCR-engineered T cells using peripheral blood from HLA-matched healthy donors, requiring only two weeks from T cell stimulation with neoantigen-loaded dendritic cells to TCR identification. Among seven ovarian tumors analyzed, three neoantigen-specific TCRs were successfully identified and validated, with TCR-engineered T cells demonstrating antigen dose-dependent cytotoxic activity. Critically, this study revealed that one neoantigen-specific TCR exhibited cross-reactivity against the corresponding wild-type peptide, underscoring the importance of rigorous validation to avoid severe immune-related adverse events [36–39]. 

In a remarkable clinical case series, Hung et al. reported two patients with heavily burdened metastatic ovarian cancer who achieved substantial tumor regression following personalized neoantigen-based T cell therapy. Neoantigen peptides were designed from tumor-specific somatic mutations and their predicted affinity for HLA molecules, then employed to stimulate T lymphocytes via co-culture with antigen-loaded dendritic cells for in vitro expansion. After treatment, both patients showed a substantial decline in tumor marker levels and significant tumor regression; notably, one patient achieved recurrent tumor responses following additional infusions of T cells targeting newly identified neoantigens. Transcriptomic profiling demonstrated an expansion of neoantigen-reactive cytotoxic T cells with a broad, polyclonal TCR repertoire in peripheral blood, along with increased expression of cytolytic proteins and inflammatory cytokines. Importantly, specific TCR clonotypes recognizing neoantigens remained detectable for months post-therapy, suggesting durable immune memory formation. Collectively, these findings support the potential of individualized neoantigen-targeted T-cell therapy to generate potent and sustained antitumor immunity in ovarian cancer [36–39]. 

##### Tumor-infiltrating lymphocyte therapy

The accumulation of tumor-infiltrating lymphocytes in ovarian cancer serves as a powerful prognostic indicator, with higher densities of CD8 + TILs consistently correlating with improved overall survival and progression-free survival across multiple independent cohorts. This favorable prognostic impact suggests that ovarian cancer is inherently immunogenic and that effective mobilization of endogenous TIL activity could translate into therapeutic benefit. However, many TILs within ovarian tumors exhibit markers of exhaustion and dysfunction, with upregulation of inhibitory receptors such as PD-1, TIM-3, LAG-3, and TIGIT, reduced production of effector cytokines including IFN-γ and TNF-α, and impaired cytolytic capacity. The presence of regulatory T cells within the TIL compartment further contributes to immunosuppression by secreting IL-10 and TGF-β and expressing CTLA-4. Despite these inhibitory influences, tumor-reactive TILs can be identified in ovarian cancer, and strategies to enrich, expand, and activate these antigen-specific populations hold therapeutic promise [31–40]. 

##### Clinical experience with TIL therapy in ovarian cancer

Adoptive TIL therapy has achieved remarkable success in metastatic melanoma, with objective response rates of 50–72% and durable complete responses in some patients following lymphodepletion and high-dose IL-2 support. While similar success has been more elusive in ovarian cancer, several clinical trials have demonstrated feasibility and encouraging signals of activity. Andersen et al. reported results from treatment of six patients with late-stage metastatic high-grade serous ovarian cancer using a combination of ipilimumab (anti-CTLA-4) followed by surgery to harvest TILs, ex vivo TIL expansion through rapid expansion protocol (REP-TILs), infusion of REP-TILs, low-dose IL-2, and nivolumab (anti-PD-1). One patient achieved a partial response and five others experienced disease stabilization for up to 12 months. Analysis of REP-TILs demonstrated primarily activated and differentiated effector memory T cells expressing inhibitory receptors LAG-3 and PD-1, with in vitro tumor reactivity. Importantly, the data suggested that ipilimumab therapy prior to TIL harvest improved T cell fold expansion during production, increased CD8 + T cell tumor reactivity, and favorably affected T cell phenotype. The combination of checkpoint inhibitors with adoptive cell therapy proved feasible and safe [31–40]. 

In a Dutch phase I/II study (NCT04072263), patients with epithelial ovarian cancer were treated with autologous TIL therapy combined with platinum-based chemotherapy. At data cutoff, the 14 patients who completed one full course of TIL therapy exhibited notably positive clinical outcomes, with the majority achieving objective tumor responses (complete or partial) and the rest maintaining stable disease, translating into high response rates and full disease control within six months. The median platinum-free duration was around 6.5 months, and progression-free survival reached 10.7 months. Importantly, this therapeutic combination did not impact circulating cytokines essential for T-cell maintenance, such as IL-7, IL-15, and IL-21. Early inclusion of interferon-α led to considerable hematologic adverse effects, but its omission resulted in a safety profile consistent with platinum chemotherapy alone. In summary, administering TIL therapy in conjunction with chemotherapy—especially within an optimal post-chemotherapy window—appears to be a practical, well-tolerated approach with substantial antitumor efficacy [41]. A comprehensive summary of the representative clinical trials evaluating engineered T-lymphocyte therapies in patients with high-grade serous ovarian carcinoma, including study design, therapeutic targets, treatment characteristics, and major clinical outcomes, is presented in (Table 1).

**Table 1:** Summary of Representative Clinical Trials of Engineered T-Lymphocyte Therapies in High-Grade Serous Ovarian Carcinoma

Target / Antigen	Platform	Clinical Trial / Phase	Patient Population	Key Efficacy Outcomes	Major Toxicities
Mesothelin (MSLN)	CAR-T	Phase I (University of Pennsylvania)	Mesothelin-positive advanced solid tumors including ovarian cancer	Disease control in 8/14 patients; no confirmed CR/PR; PFS approximately 4–6 months in ovarian cancer cohorts	Grade 3 CRS, respiratory toxicity at higher doses; no significant neurotoxicity
Mesothelin (MSLN) + IL-7/CCL19	CAR-T	Early Phase I	Recurrent ovarian cancer	Safe intraperitoneal administration; no meaningful objective responses	Mainly grade 1–2 adverse events; no severe CRS
Mesothelin (MSLN) DAP-CAR	CAR-T	Early clinical study	Advanced ovarian cancer	Partial responses and disease stabilization observed; encouraging preliminary efficacy	Favorable safety profile; manageable CRS
Folate Receptor-α (FRα)	CAR-T	NCT01567891, Phase I	Recurrent HGSOC with high FRα expression	Feasible and safe; no significant reduction in tumor burden reported to date	Mild infusion-related adverse events; no dose-limiting toxicity reported
FSH Receptor (FSHR)	CAR-T	Phase I dose-escalation	Advanced ovarian cancer	Stable disease in one patient for > 1 year; dose escalation completed without DLT	No dose-limiting toxicity observed
MUC16 (CA125)	CAR-T	NCT06469281, Phase I	Advanced epithelial ovarian cancer	Ongoing; efficacy results pending	Safety under evaluation
MUC16ecto + IL-12	CAR-T	Phase I	Recurrent ovarian cancer	Intraperitoneal delivery feasible; limited clinical responses	Acceptable safety profile
B7-H3 (CD276)	CAR-T	Stanford Phase I	Platinum-resistant ovarian cancer	Preliminary disease responses observed in first treated patients	Manageable toxicity
B7-H3 (CD276)	CAR-T	NCT06305299, Phase I	Platinum-resistant epithelial ovarian cancer	Ongoing dose-escalation study	Safety under investigation; inducible caspase-9 safety switch incorporated
Mesothelin (MSLN)	TCR-T	Preclinical translational study	HLA-A2 + HGSOC	Prolonged survival in murine models (112 vs. 77 days); enhanced tumor infiltration	No on-target/off-tumor toxicity observed
Personalized Neoantigens	TCR-T	Investigator-initiated clinical study	Metastatic ovarian cancer	Significant tumor regression; durable neoantigen-specific T-cell persistence; reduction of tumor markers	No severe immune-related toxicity reported
Tumor-Infiltrating Lymphocytes (TILs)	TIL	Andersen et al., Pilot study	Metastatic HGSOC	1 partial response; 5 stable disease; disease control up to 12 months	Well tolerated with low-dose IL-2 and checkpoint inhibitors
Tumor-Infiltrating Lymphocytes (TILs)	TIL	NCT04072263, Phase I/II	Epithelial ovarian cancer	High disease control rate; median PFS 10.7 months; platinum-free interval 6.5 months	Hematologic toxicity mainly related to chemotherapy; acceptable overall safety

*Abbreviations*: *CAR-T* Chimeric antigen receptor T-cell, *TCR-T* T-cell receptor-engineered T-cell, *TIL* Tumor-infiltrating lymphocyte, *HGSOC* High-grade serous ovarian carcinoma, *ORR* Objective response rate, *PFS* Progression-free survival, *OS* Overall survival, *CR* Complete response, *PR* Partial response, *SD* Stable Disease, *DLT* Dose-limiting toxicity, *CRS* Cytokine release syndrome, *IL* Interleukin, *MSLN* Mesothelin, *FRα* Folate receptor alpha, *MUC16* Mucin 16,* B7-H3* B7 homolog 3, *FSHR* Follicle-stimulating hormone receptor,* HLA* Human leukocyte antigen

### Engineered T cells in apoptosis induction

Engineered T-cell therapies, including chimeric antigen receptor T-cell (CAR-T) and T-cell receptor-engineered T-cell (TCR-T) platforms, have emerged as transformative immunotherapeutic strategies capable of enhancing apoptosis-mediated tumor eradication beyond the capacity of endogenous antitumor immunity. Unlike natural CD8⁺ T lymphocytes, which require coordinated antigen presentation, costimulatory signaling, and cytokine-mediated activation to achieve full effector function, engineered T cells are genetically modified to express high-affinity tumor-targeting receptors that enable rapid and sustained activation upon antigen recognition. This engineering strategy circumvents the limitations imposed by the low frequency and suboptimal avidity of naturally occurring tumor-reactive T-cell clones, thereby enhancing the magnitude and specificity of antitumor responses. CAR-T cells possess a unique advantage in that they recognize tumor-associated surface antigens independently of major histocompatibility complex (MHC) presentation, thereby overcoming a common mechanism of tumor immune escape involving MHC downregulation or defective antigen-processing pathways. In contrast, TCR-T cells exploit the physiological sensitivity of the T-cell receptor to recognize intracellular tumor-derived peptides presented by human leukocyte antigen (HLA) molecules, substantially expanding the repertoire of targetable antigens to include neoantigens, oncogenic driver mutations, and other intracellular proteins inaccessible to antibody-based therapies. Together, these complementary approaches provide broad opportunities for targeting diverse tumor antigens while minimizing immune evasion [42]. 

The enhanced therapeutic efficacy of engineered T cells is further supported by the incorporation of intracellular signaling and costimulatory domains that amplify T-cell activation, proliferation, persistence, and effector function. In CAR-T cells, signaling motifs such as CD3ζ combined with costimulatory domains including CD28 or 4-1BB generate potent activation cascades that promote cytokine production, clonal expansion, and sustained cytotoxicity. These modifications partially overcome the functional impairment typically induced by chronic antigen exposure within the tumor microenvironment. Consequently, engineered T cells exhibit greater resistance to exhaustion and maintain antitumor activity despite prolonged stimulation by persistent tumor antigens. Following antigen engagement, engineered T cells induce apoptosis through mechanisms analogous to, but often more potent than, those employed by endogenous cytotoxic T lymphocytes. These include perforin- and granzyme-mediated cytotoxicity, in which perforin facilitates granzyme entry into target cells to activate caspase-dependent apoptotic pathways, as well as Fas ligand (FasL)-mediated death receptor signaling that triggers extrinsic apoptosis. Enhanced receptor avidity and sustained activation increase the efficiency of cytotoxic granule release and serial killing capacity, enabling individual engineered T cells to eliminate multiple tumor cells sequentially. In addition, the secretion of proinflammatory cytokines such as interferon-γ (IFN-γ) and tumor necrosis factor-α (TNF-α) further amplifies antitumor immunity by promoting immune-cell recruitment, macrophage activation, and inhibition of tumor growth and angiogenesis [42]. 

**Fig. 5:**
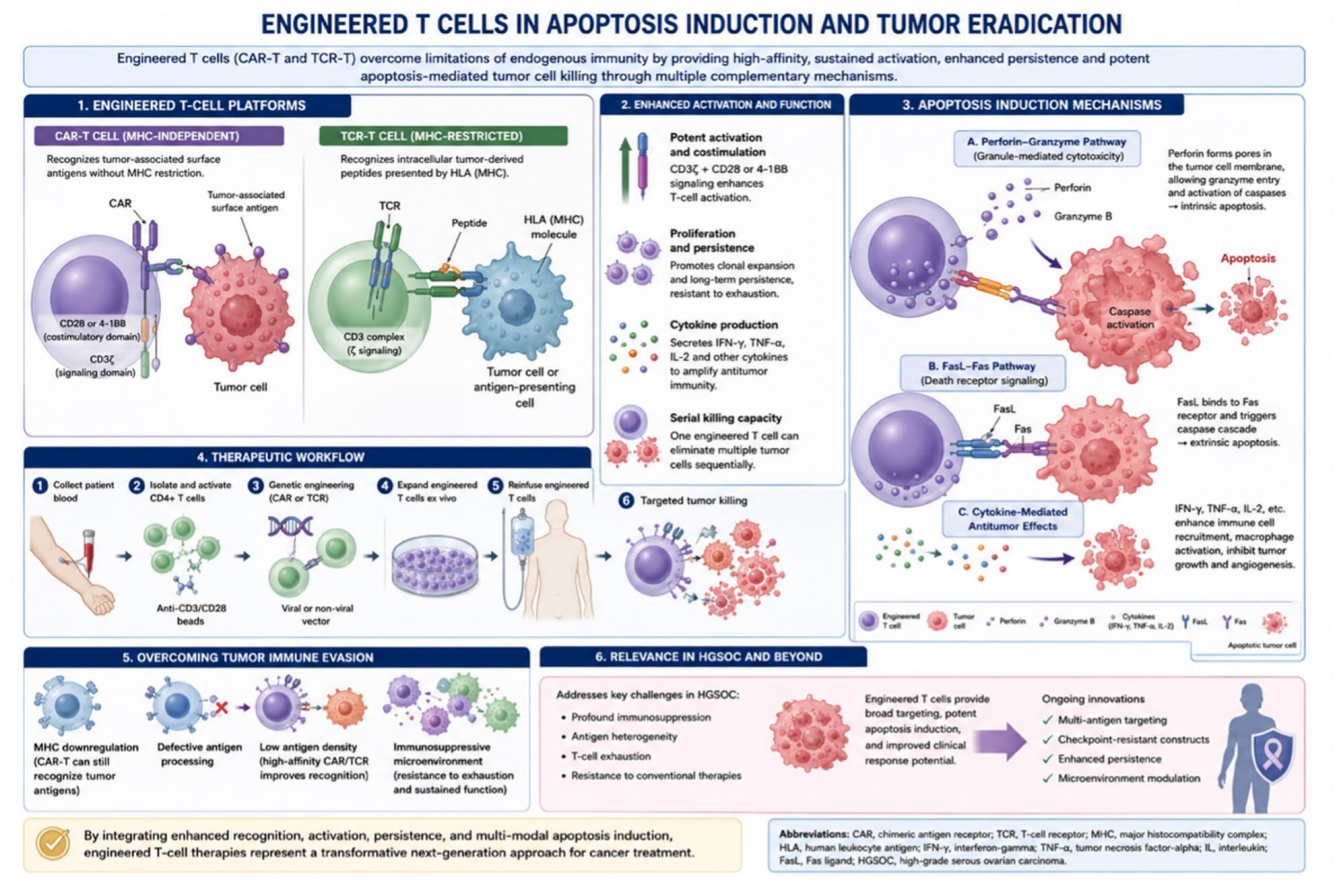
Engineered T-cell–mediated apoptosis induction and antitumor mechanisms. Schematic illustration depicting the complementary mechanisms of CAR-T and TCR-T cell therapies, including antigen recognition, intracellular signaling, cytotoxic effector functions, apoptosis induction pathways, and strategies for overcoming tumor immune evasion. Created with AI-assisted image generation under author supervision and scientifically reviewed by the authors

These properties are particularly relevant in high-grade serous ovarian carcinoma (HGSOC), where profound immunosuppression, antigen heterogeneity, T-cell exhaustion, and resistance to conventional therapies frequently limit durable clinical responses. By combining enhanced antigen recognition, integrated activation signaling, prolonged persistence, and potent apoptosis-inducing mechanisms, engineered T-cell therapies have the potential to overcome key barriers to effective antitumor immunity. Although challenges related to tumor heterogeneity, immune escape, and the immunosuppressive tumor microenvironment remain, ongoing advances in receptor engineering, multi-antigen targeting, checkpoint-resistant constructs, and microenvironment-modulating strategies continue to improve the therapeutic potential of CAR-T and TCR-T platforms. Collectively, these innovations position engineered T-cell therapy as a promising next-generation approach for the treatment of ovarian cancer and other malignancies characterized by immune resistance and therapeutic refractoriness (Fig. 5) [42].

#### Comparative apoptotic activity of natural CD8⁺ T Cells and Engineered CAR-T/TCR-T cells

Natural CD8⁺ cytotoxic T lymphocytes (CTLs) constitute the principal effector cells of adaptive antitumor immunity, eliminating malignant cells through perforin–granzyme exocytosis and Fas/Fas ligand (FasL)-mediated apoptosis. However, their cytotoxic activity depends on efficient antigen presentation through major histocompatibility complex class I (MHC-I), adequate co-stimulatory signaling, and sustained cytokine support. Within the tumor microenvironment (TME), chronic antigen exposure, inhibitory checkpoint signaling, and metabolic stress progressively impair CTL function, resulting in delayed cytotoxic kinetics, diminished serial killing capacity, reduced cytokine secretion, and eventual T-cell exhaustion. In contrast, engineered T cells—including chimeric antigen receptor T (CAR-T) cells and T-cell receptor-engineered T (TCR-T) cells—are specifically designed to enhance both the magnitude and persistence of apoptosis induction. CAR-T cells recognize surface tumor antigens independently of MHC presentation, thereby overcoming one of the most common immune escape mechanisms employed by ovarian cancer cells, namely MHC-I downregulation. Meanwhile, TCR-T cells maintain physiological peptide-MHC recognition while expressing affinity-enhanced T-cell receptors capable of recognizing tumor-associated antigens with substantially greater sensitivity than endogenous T cells. Furthermore, incorporation of intracellular signaling domains such as CD28, 4−1BB, or ICOS amplifies downstream activation of ZAP70, PI3K/AKT, NF-κB, and MAPK pathways, promoting rapid proliferation, prolonged persistence, enhanced cytokine production, and superior serial tumor-cell killing. Consequently, engineered T cells exhibit faster apoptotic kinetics, greater cytotoxic potency, and sustained antitumor activity compared with conventional CD8⁺ T lymphocytes [42]. 

#### Tumor resistance mechanisms against T-cell-induced apoptosis

Despite enhanced cytotoxicity, ovarian cancer cells possess multiple molecular mechanisms that attenuate apoptosis and limit the efficacy of engineered T-cell therapies. One of the most prominent mechanisms involves overexpression of anti-apoptotic members of the B-cell lymphoma−2 (Bcl−2) protein family, including Bcl−2, Bcl-xL, and Mcl−1, which inhibit mitochondrial outer membrane permeabilization and prevent cytochrome-c release, thereby blocking activation of caspase−9 and downstream executioner caspases. Elevated expression of these proteins has been associated with platinum resistance and poor prognosis in high-grade serous ovarian carcinoma. Another important resistance mechanism is the upregulation of cellular FLICE-like inhibitory protein (c-FLIP), which competitively inhibits caspase−8 activation within the death-inducing signaling complex (DISC). Consequently, tumor cells become resistant to Fas/FasL-mediated apoptosis despite successful immune synapse formation. In parallel, ovarian cancer cells may express intracellular serine protease inhibitors (serpins), particularly SerpinB9, which directly neutralizes granzyme B after its entry into the cytoplasm, thereby preventing perforin/granzyme-mediated apoptosis. Additional resistance mechanisms include impaired death receptor expression, activation of PI3K/AKT and NF-κB survival pathways, mutations affecting p53 signaling, and persistent immunosuppressive cytokines such as transforming growth factor-β (TGF-β) and interleukin−10 (IL−10), all of which collectively reduce susceptibility to engineered T-cell-mediated cytotoxicity [42]. 

#### Next-generation engineering strategies to enhance apoptosis induction

**Fig. 6:**
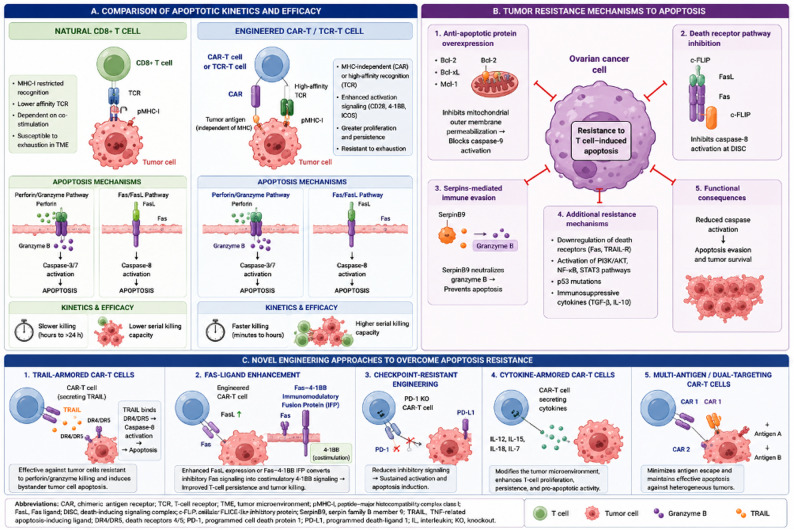
Comparison of apoptotic mechanisms, tumor resistance pathways, and next-generation engineering strategies in T-cell–based immunotherapy for high-grade serous ovarian carcinoma. Schematic illustration comparing the apoptotic kinetics and cytotoxic efficacy of natural CD8⁺ T cells and engineered CAR-T/TCR-T cells. The figure summarizes the principal mechanisms of T-cell-mediated apoptosis through perforin–granzyme release and Fas/Fas ligand signaling, major molecular mechanisms of tumor resistance to apoptosis—including overexpression of anti-apoptotic Bcl−2 family proteins, c-FLIP-mediated inhibition of death receptor signaling, SerpinB9-mediated granzyme B neutralization, and activation of prosurvival pathways—and emerging engineering strategies designed to overcome these barriers. These approaches include TRAIL-armored CAR-T cells, Fas-ligand/Fas–4−1BB immunomodulatory fusion proteins, checkpoint-resistant CAR-T cells, cytokine-armored CAR-T cells, and multi-antigen targeting platforms that enhance apoptosis induction, improve T-cell persistence, and reduce tumor immune escape. This figure was conceptually designed by the authors and generated with the assistance of artificial intelligence (AI)-based image generation tools, followed by author review, scientific refinement, and verification for biological accuracy

Recent advances in cellular engineering have focused on overcoming apoptotic resistance by equipping engineered T cells with additional pro-apoptotic functions. One promising strategy involves TRAIL-armored CAR-T cells, which constitutively express tumor necrosis factor-related apoptosis-inducing ligand (TRAIL). TRAIL binds death receptors DR4 and DR5 expressed on tumor cells, activating the extrinsic apoptotic pathway independently of perforin-mediated cytotoxicity. Preclinical studies have demonstrated that TRAIL-secreting CAR-T cells effectively eradicate tumor cells resistant to conventional CAR-mediated killing while simultaneously inducing bystander tumor cell apoptosis. Similarly, Fas-ligand-enhanced CAR-T cells have been engineered to increase FasL expression or to incorporate Fas–4−1BB immunomodulatory fusion proteins (IFPs). Rather than transmitting apoptotic signals into the T cell upon Fas engagement, these constructs convert inhibitory Fas signaling into costimulatory 4−1BB activation, thereby enhancing T-cell proliferation, persistence, and resistance to activation-induced cell death. This strategy has shown encouraging preclinical efficacy in ovarian cancer models by significantly prolonging T-cell survival while maintaining potent antitumor activity. Additional next-generation platforms include dominant-negative TGF-β receptors, cytokine-armored CAR-T cells secreting IL−12, IL−15, or IL−18, checkpoint-resistant CAR-T cells lacking PD−1 expression through CRISPR-mediated editing, and multi-antigen CAR constructs designed to minimize antigen escape. Collectively, these innovations aim not only to enhance direct apoptosis induction but also to remodel the immunosuppressive tumor microenvironment, thereby achieving more durable and clinically meaningful antitumor responses. Collectively, engineered T-cell therapies induce apoptosis through multiple complementary mechanisms that exceed the cytotoxic potential of endogenous CD8⁺ T lymphocytes. Nevertheless, intrinsic tumor resistance mediated by anti-apoptotic signaling pathways and the immunosuppressive tumor microenvironment continues to limit therapeutic efficacy in high-grade serous ovarian carcinoma. Emerging next-generation engineering strategies—including apoptosis-armored CAR constructs, immune checkpoint-resistant T cells, and multifunctional cytokine-secreting platforms—provide promising approaches to overcome these barriers and may substantially improve the clinical effectiveness of adoptive cellular immunotherapy in ovarian cancer (Fig. 6) [42].

## Conclusions

Engineered T-cell therapies, including chimeric antigen receptor T-cell (CAR-T) and T-cell receptor-engineered T-cell (TCR-T) platforms, represent a promising advancement in the treatment of high-grade serous ovarian carcinoma (HGSOC), a malignancy characterized by profound molecular heterogeneity, therapeutic resistance, and an immunosuppressive tumor microenvironment. By enhancing antigen recognition, amplifying intracellular activation signaling, and sustaining cytotoxic effector functions, engineered T cells overcome several limitations of endogenous antitumor immunity. CAR-T cells provide major histocompatibility complex (MHC)-independent targeting of tumor-associated surface antigens, whereas TCR-T cells extend therapeutic applicability to intracellular antigens and neoantigens presented through human leukocyte antigen (HLA) molecules. Both approaches induce potent tumor cell apoptosis through perforin–granzyme release, Fas–Fas ligand signaling, and cytokine-mediated antitumor effects.

Despite encouraging preclinical and early clinical outcomes, several challenges continue to limit the efficacy of engineered T-cell therapies in ovarian cancer, including antigen heterogeneity, tumor antigen escape, inadequate T-cell trafficking, functional exhaustion, and the highly immunosuppressive tumor microenvironment. Advances in multi-antigen targeting, checkpoint-resistant constructs, cytokine-armored CARs, neoantigen-specific TCRs, and combination strategies with immune checkpoint inhibitors or conventional therapies are actively being developed to address these barriers. In parallel, tumor-infiltrating lymphocyte (TIL)-based approaches further support the therapeutic potential of adoptive cellular immunotherapy in ovarian cancer.

Collectively, current evidence suggests that engineered T-cell therapies have the capacity to transform the management of HGSOC by providing highly specific, durable, and adaptable antitumor immune responses. Continued optimization of target selection, cellular engineering, and microenvironmental modulation, together with well-designed clinical trials, will be essential to fully realize their therapeutic potential and establish engineered T-cell therapy as a cornerstone of precision immuno-oncology for ovarian cancer.

## Data Availability

No datasets were generated or analysed during the current study.
